# BCL-2 Inhibition via Venetoclax at ART Initiation Induces Long-Term Reduction of the Intact SIV Reservoir

**DOI:** 10.21203/rs.3.rs-7060088/v1

**Published:** 2025-07-14

**Authors:** Tomas Raul Wiche Salinas, Justin Harper, Claire Deleage, Kevin Nguyen, James Auger, Hannah R. Flores, Amelia C. Wilkes, Rachelle L. Stammen, Jennifer S. Wood, Gregory K. Tharp, Steven E. Bosinger, Mackenzie L. Cottrell, Emek Kose, Taina T. Immonen, Jeffrey D. Lifson, Gregory M. Laird, Brandon F. Keele, R. Brad Jones, Andrew D. Badley, Guido Silvestri, Deanna A. Kulpa, Mirko Paiardini

**Affiliations:** 1 Division of Microbiology and Immunology, Emory National Primate Research Center, Emory University, Atlanta, GA, USA; 2 AIDS and Cancer Virus Program, Frederick National Laboratory for Cancer Research, Frederick, MD, USA.; 3 Division of Animal Resources, Emory National Primate Research Center, Emory University, Atlanta, GA, USA; 4 Department of Pathology and Laboratory Medicine, Emory University School of Medicine, Atlanta, GA, USA; 5 Division of Pharmacotherapy and Experimental Therapeutics, University of North Carolina UNC Eshelman School of Pharmacy, Chapel Hill, North Carolina, USA.; 6 Accelevir Diagnostics, Baltimore, Maryland, USA.; 7 Infectious Diseases Division, Department of Medicine, Weill Cornell Medical College, New York, NY, USA.; 8 Department of Microbiology and Immunology, Weill Cornell Graduate School of Medical Sciences, New York, NY, USA.; 9 Division of Infectious Diseases, Mayo Clinic, Rochester, Minnesota, USA; 10 Department of Molecular Medicine, Mayo Clinic, Rochester, Minnesota, USA

## Abstract

The anti-apoptotic molecule BCL-2 favors the maintenance of the CD4^+^ T-cell reservoir during Human Immunodeficiency Virus (HIV) infection. We investigated directly in-vivo whether BCL-2 inhibition using venetoclax at the initiation of antiretroviral therapy (ART) would reduce the size of the viral reservoir. Twenty-four SIV_mac239_-infected rhesus macaques (RMs) initiated ART at day 14 post-infection (p.i.), alone or with a 10-day treatment with venetoclax or venetoclax plus CD8α depletion, and followed up to day 294 p.i. A rapid, statistically significant, and sustained reduction in the intact SIV reservoir was observed in venetoclax-treated RMs in blood and lymph nodes (LNs). This reduction was driven by reduced survival and depletion of CD4^+^T-cell subsets that critically contribute to the reservoir. CD4^+^ T-cells that persisted after venetoclax treatment exhibited elevated per-cell levels of BCL-2, reduced expression of pro-apoptotic molecules such as PUMA, increased expression of additional anti-apoptotic molecules, including BCL-xL, and a partial reduction in apoptotic sensitivity in ex vivo assays. These findings provide mechanistic insights for the venetoclax-induced pro-cell death changes in CD4+ T-cells, support the rationale for extended venetoclax dosing, and suggest that combining BCL-2 inhibition with agents targeting additional anti-apoptotic molecules can enhance clearance of the viral reservoir in HIV cure strategies.

ART effectively suppresses HIV replication in most people living with HIV (PLWH). Although there is a decay in the HIV reservoir following ART initiation (ARTi), the virus persists even after long-term treatment. This persistence occurs largely because HIV can remain either in a latent state or transcriptionally active in long-lived CD4^+^ T-cells, whose survival is regulated by anti-apoptotic proteins of the BCL-2 family^1^. Consistently, the HIV reservoir is enriched in CD4^+^ T-cells expressing survival-promoting markers, such as IL-7R and OX40, which upregulate the anti-apoptotic protein BCL-2^2,3^. Furthermore, the reservoir is particularly abundant in BCL-2^+^ CD4^+^ T-cells and on CD4^+^ T-cells with transcriptional signatures of enhanced survival^4,5^.

Mechanistically, HIV protease induces the cleavage of procaspase-8, producing Casp8p41, which sensitizes cells to apoptosis. However, infected cells over-express BCL-2, which binds and inactivates Casp8p41, thereby preventing cell death. Inhibition of BCL-2 restores the pro-apoptotic activity of Casp8p41, driving infected cells towards apoptosis^6^. Additionally, BCL-2 prevents cytotoxic killing mediated by CD8^+^ T-cells and NK cells by sequestering Bid and preventing the mitochondrial membrane permeabilization^4,7^. Consistently, combining BCL-2 inhibition with latency-reversing agents (LRAs) and HIV-specific cytolytic cells effectively depleted the HIV reservoir ex vivo^4^, and enhanced the clearance of HIV-infected cells by NK cells in vitro^7^. Thus, high levels of BCL-2 and its mechanisms of action described above enable the HIV reservoir to evade both the cytopathic effects of HIV and the cytolytic activities of CD8^+^ T-cells and NK cells, promoting its long-term persistence.

The impact of BCL-2 inhibition using venetoclax, a clinically approved drug for multiple cancer conditions^8^ has been evaluated in humanized mouse models of HIV infection. In an acute infection model, humanized mice infected with HIV received venetoclax from one day before infection and for 10 weeks, resulting in reduced plasma viremia beginning at week 6 p.i. and fewer mice with detectable intact proviral HIV DNA compared to the untreated, control group^4^. In another study, HIV-infected mice that achieved viral suppression with ART started venetoclax for 6 weeks. These treated mice exhibited a short (up to two weeks) but significant delay in viral rebound following ART discontinuation^9^. Altogether, these findings highlight the potential of BCL-2 inhibition to limit viral persistence and provide rationale for testing this intervention in the most clinically relevant nonhuman primate (NHP) models for HIV infection.

In the present study, we conducted the first NHP evaluation of short-term BCL-2 inhibition using venetoclax in SIV-infected RMs. Venetoclax was initiated concurrently with ART to enhance the death of infected cells, by both the cytopathic effects of SIV and the cytolytic activity of CD8^+^ T-cells, which would otherwise become part of the long-lived reservoir. We previously demonstrated that CD8α depletion before early ARTi results in a slower decline in viremia without altering the frequency of CD4^+^ T-cells harboring intact provirus^10^. Therefore, we combined venetoclax with the anti-CD8α depleting antibody MT807R1 to further expose SIV-infected CD4^+^ T-cells to SIV-induced cytopathic effects. This design enables us to determine whether prolonged virus production during ART favors venetoclax-mediated reduction in the viral reservoir and if CD8 T-cells are critical for the anti-reservoir activity of venetoclax. Our findings support a role for BCL-2 inhibition at ARTi in promoting the elimination of SIV-infected CD4^+^ T-cells that will, otherwise, sustain the persistent SIV reservoir. This study provides a strong rationale for targeting BCL-2 in HIV-cure related strategies and identifies additional anti-apoptotic pathways to target in combination with venetoclax for reservoir eradication.

## Results

### Study Design

**Fig 1.**
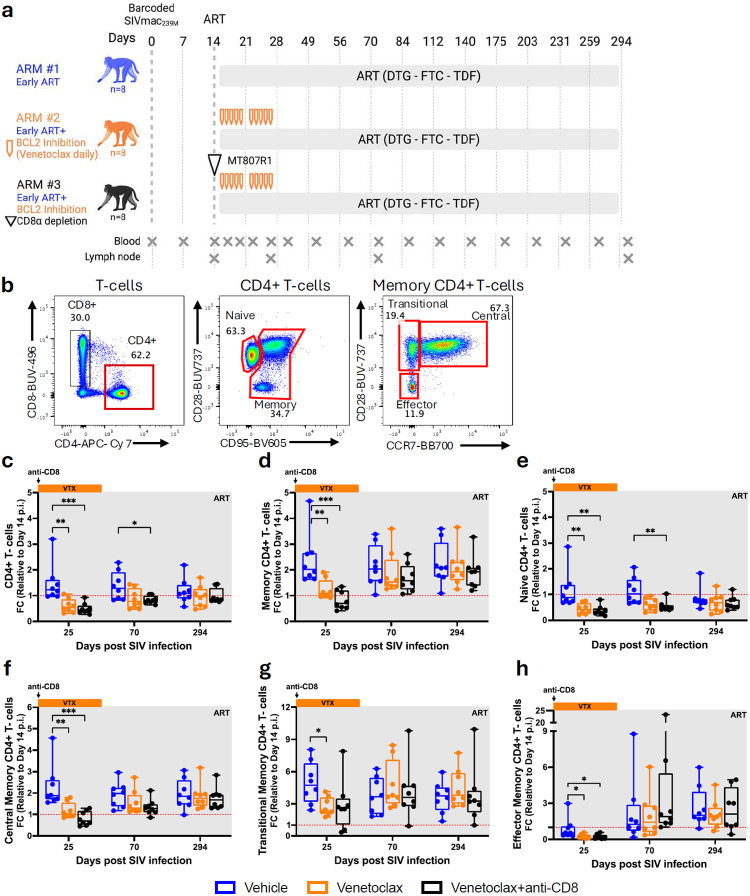
BCL-2 inhibition via venetoclax depletes CD4+ T cell subsets associated with viral persistence. a, Schematic for the study design. RMs were infected i.v. with SIVmac239 and initiated on ART at day 14 p.i. Venetoclax (10 daily doses) and/or anti-CD8α antibody (1 dose) were started at ARTi until day 25 p.i., with follow-up through day 294 p.i. Blood and LNs samples were collected at the indicated time points. b, Flow cytometry representative staining and gating strategy used to identify CD4^+^ T cells subsets. c-h, Fold change of CD4^+^ T cells counts relative to baseline in blood, including total (c), memory (d), naïve (e), central memory (f), transitional memory (g), and effector memory (h) CD4^+^ T cells. RMs are color-coded and grouped based on treatment arm, with population sizes indicated for all analyses: vehicle (n = 8), blue; venetoclax (n = 8), orange; venetoclax + anti-CD8 (n = 8), black. All data are presented as median ± 25th and 75th percentile and were analyzed using a two-sided Mann–Whitney U test. *P ≤ 0.05, **P < 0.01, ***P < 0.001, ****P < 0.0001.

Twenty-four Indian RMs were intravenously (i.v.) infected with 10,000 infectious units (IU) of barcoded SIVmac239M and initiated triple-combination ART, consisting of tenofovir disoproxil fumarate (TDF), dolutegravir (DTG), and emtricitabine (FTC), at day 14 p.i. Based on their age and viral load before starting ART, RMs were evenly distributed (n=8) between the three experimental arms: vehicle, venetoclax, or venetoclax plus anti-CD8α depletion (Fig. 1a). In the vehicle arm, the RMs received ART alone. In the venetoclax arm, RMs received ten doses of venetoclax (daily, Monday-Friday, for two consecutive weeks) starting at ARTi, whereas in the venetoclax plus anti-CD8α arm, RMs received the same regimen of venetoclax (with the exception of one animal that received eight doses) plus a single dose of the anti-CD8α-depleting antibody MT807R1 at ARTi. RMs were followed up to day 294 p.i. Venetoclax regimen was optimized with a pilot study where we compared plasma concentration (Supplementary Fig.1a) and total exposure of venetoclax (AUC, Supplementary Fig.1b) and CD4^+^ T-cell levels, both as absolute counts (Supplementary Fig.1c) or fold change from baseline (Supplementary Fig.1d) in SIV-uninfected RMs receiving venetoclax subcutaneously (SC), with an oral gavage (OG), or OG plus ketoconazole. Pharmacokinetics and, more importantly, immunological effects were comparable between the tested SC and OG routes, as evidenced by similar depletion of circulating CD4^+^ T-cells (Supplementary Fig.1). Based on those data, venetoclax was administered either subcutaneously (SC, at 10 mg/kg or 20 mg/kg) or, due to abscess formation following repeated SC doses, as a combination of SC (20 mg/kg for the first two doses for each of the two 5-days treatment cycles) and OG (400 mg venclexta, the FDA-approved formulation of venetoclax, for the final three doses for each of the two 5-days treatment cycle) co-administered with the CYP3A inhibitor ketoconazole (10 mg/kg).

### Impact of BCL-2 inhibition on CD4^+^ T cell dynamics at ARTi

**Extended Data Fig. 1.**
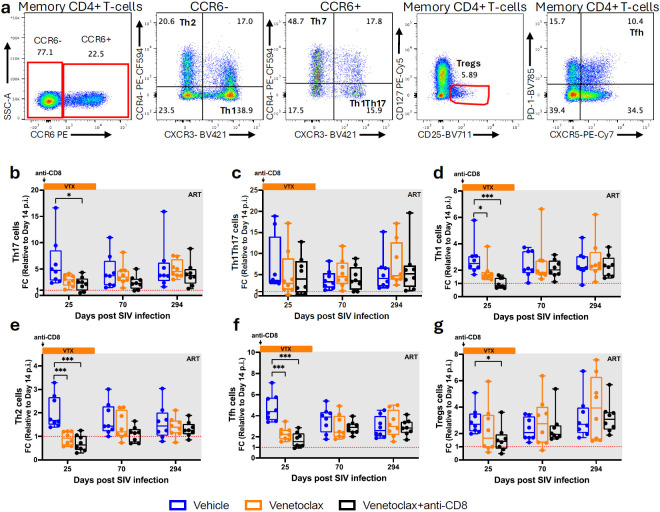
Venetoclax effectively targets functional CD4^+^ T helper cells subsets. **a,** Representative gating strategy used to identify CD4^+^ T helper functional subsets. b–g, Fold change of Th17 (b), Th1Th17 (c), Th1 (d), Th2 (e), Tfh (f), and Tregs (g) cells counts relative to baseline in blood. RMs are color-coded and grouped based on treatment arm, with population sizes indicated for all analyses: vehicle (n = 8), blue; venetoclax (n = 8), orange; venetoclax + anti-CD8 (n = 8), black. All data are presented as median ± 25^th^ and 75^th^ percentile and were analyzed using a two-sided Mann–Whitney U test. *P ≤ 0.05, **P < 0.01, ***P < 0.001, ****P < 0.0001.

To determine the effects of venetoclax-induced BCL-2 inhibition on CD4^+^ T-cells, we measured their absolute counts and main differentiation subsets using flow cytometry (Fig. 1b) at day 14 p.i., as the pre-ART and pre-venetoclax baseline; day 25 p.i., corresponding to the end of venetoclax treatment; and during follow-up at days 70 and 294 p.i. In vehicle-treated RMs, peripheral blood CD4^+^ T-cell counts increased at day 25 p.i. relative to day 14, as expected with initiation of ART (Fig. 1c). In contrast, RMs treated with venetoclax alone or in combination with anti-CD8α (hereafter referred to as venetoclax-treated RMs) showed lower CD4^+^ T cell counts at day 25 p.i. compared to their baseline values and the vehicle-treated RMs (Fig. 1c; Supplementary Fig. 1a). The reduction in CD4^+^ T-cells in the venetoclax-treated RMs was transient, with their levels no longer different from control animals at the latest time points. The finding that venetoclax-treated RMs have reduced CD4^+^ T-cell levels during treatment with a long-term reconstitution similar to the levels found in control RMs is consistent for all the main CD4^+^ T-cell subsets, including naïve (Fig. 1e, CD28^+^CD95^−^), memory (Fig. 1f, CD95^+^), and the memory subsets preferentially associated with viral persistence, such as central (CD28^+^CCR7^+^), transitional (CD28^+^CCR7^−^), and effector memory (CD28^−^CCR7^−^) (Fig. 1 f-h). Consistently, in a cross-sectional analysis at day 25 p.i., venetoclax-treated RMs exhibited lower counts across CD4^+^ T-cell subsets compared to vehicle-treated RMs (Supplementary Fig. 2a–f). To further characterize the immunological impact of venetoclax, we determined the levels of T helper (T_H_) functional subsets. This analysis showed that vehicle-treated RMs displayed increases in memory T_H_17 (CCR6^+^CCR4^+^CXCR3^−^), T_H_1T_H_17 (CCR6^+^CCR4^−^CXCR3^+^), T_H_1 (CCR6^−^CCR4^−^CXCR3^+^), T_H_2 (CCR6^−^CCR4^+^CXCR3^−^), circulating T follicular helper (T_FH_,CXCR5^+^PD-1^+^), and regulatory T (T_REG_, CD127^−^CD25^+^) cells at day 25 p.i. relative to day 14 p.i., as expected with ARTi. These increases were attenuated in venetoclax-treated RMs (Extended Fig. 1a-g). Cross-sectionally, most T_H_ subsets also exhibited lower counts in venetoclax-treated RMs compared to vehicle controls.

These findings demonstrate that BCL-2 inhibition via venetoclax effectively targets multiple CD4^+^ T-cell subsets critically contributing to HIV/SIV persistence.

### BCL-2 inhibition upregulates pro cell-death genes and downregulates survival genes in CD4^+^ T-cells

**Fig. 2.**
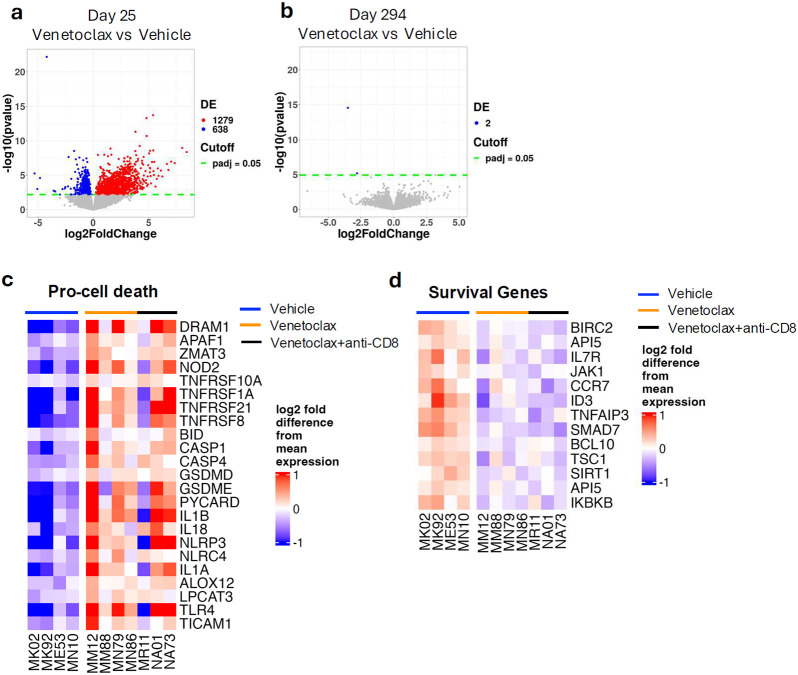
BCL-2 inhibition upregulates genes promoting cell death and downregulates survival genes in CD4^+^ T-cells. a, b Volcano plot showing differentially expressed genes (DEGs) in CD4^+^ T cells sorted from venetoclax and venetoclax + anti-CD8− treated RMs compared to vehicle-treated RM (a) at the end of venetoclax treatment (day 25 p.i.) or (b) at the end of the follow-up period (day 294 p.i.). c, d, Heatmap showing log_2_ fold difference from mean expression of representative pro-death genes (c) and survival genes (d) upregulated or downregulated, respectively, by venetoclax treatment. Analyses were performed on samples from vehicle (n = 4), venetoclax (n = 4), and venetoclax + anti-CD8 (n = 3) treated RMs. Gene selection was based on relevance to cell death pathways. Genes with an adjusted P < 0.05 were considered for heatmaps.

To investigate the effects of BCL-2 inhibition via venetoclax in SIV-infected RMs and to elucidate the mechanisms underlying the transient CD4^+^ T-cell depletion, we performed RNA sequencing on CD4^+^ T-cells sorted at the end of venetoclax treatment (day 25 p.i.) and at the end of the follow-up period (day 294 p.i.). This analysis was conducted in a subset of RMs treated with vehicle (n=4), venetoclax alone (n=4), or venetoclax plus anti-CD8α depleting antibody MT807R1 (n=3) and with a primary objective to identify changes in the expression of genes regulating cell death or survival. As for the immunological data described above, the two groups of venetoclax-treated RMs have comparable transcriptional profile. Venetoclax-treated RMs displayed extensive transcriptional changes, with 1,279 differentially expressed genes (DEGs) upregulated and 638 downregulated at the end of treatment (Fig. 2a, Data set 1). These changes were largely transient, as only two DEGs remained downregulated at the conclusion of the follow-up period (Fig. 2b).

Several transcripts associated with pathways promoting cell death were elevated in venetoclax-treated RMs (Fig. 2c). For example, genes involved in apoptosis were upregulated, including DRAM1, which inhibits the degradation of the pro-apoptotic protein BAX^11^; APAF1, a key component of the apoptosome required for caspase activation^12^; and NOD2, a direct activator of caspase-9^13^. Apoptosis-inducing receptors such as TNFRSF10A, TNFRSF1A, TNFRSF21 and TNFRSF8 were also upregulated^14-17^. Additionally, there was a trend toward increased expression of the pro-apoptotic molecule BID. Notably, genes involved in other cell death pathways were also upregulated. These included components of pyroptosis (e.g., CASP1, CASP4, GSDMD, GSDME, PYCARD, IL1B, IL18, NLRP3, NLRC4, and IL1A)^18-21^, ferroptosis(e.g., ALOX12 and LPCAT3)^22,23^, and necroptosis(e.g., TLR4, and TICAM1)^24-26^.

Conversely, we observed downregulation of genes associated with cell survival (Fig. 2d), including direct inhibitors of apoptosis such as BIRC2, which ubiquitinates caspases for proteasomal degradation^27^, and API5, which suppresses E2F1-induced apoptosis^28^. The expression of IL7R and JAK1, which promote T cell survival through upregulation of BCL-2^29^, was also reduced. Similarly, other transcripts that support T cell survival were downregulated in venetoclax-treated RMs, such as CCR7 and ID3^30,31^. TNFAIP3, a negative regulator of RIPK1-dependent apoptosis and necroptosis^32^, was also downregulated. A decrease in SMAD7, which may protect against TGF-β–mediated apoptosis^33^ and in BCL10, which is an adaptor that contributes to cell survival after DNA damage by activating NF-κB signaling^34^, was also observed.

Altogether, these findings support and provide mechanistic insights for the efiicacy of BCL-2 inhibition via venetoclax in promoting cell death by inducing extensive, yet transient, pro-cell death transcriptional changes in CD4^+^ T-cells.

### BCL-2 inhibition via venetoclax induces a rapid and prolonged reduction of the intact SIV reservoir

**Extended Data Fig 2.**
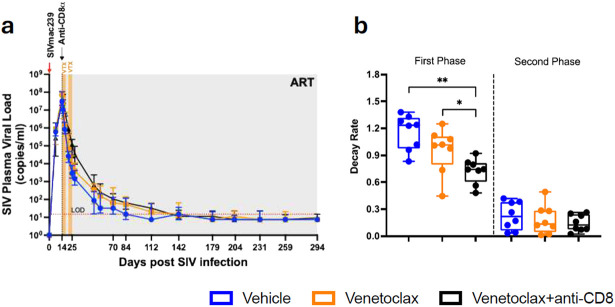
SIV plasma viral load kinetics. a, Plasma viral loads in vehicle-, venetoclax-, and venetoclax + anti-CD8–treated RMs from day 7 p.i. to day 294 p.i. b, Decay rates of plasma viral loads calculated using a biphasic decay model. RMs are color-coded and grouped based on treatment arm, with population sizes indicated for all analyses: vehicle (n = 8), blue; venetoclax (n = 8), orange; venetoclax + anti-CD8 (n = 8), black. The detection limit for plasma viral load assays was 15 copies/ml through day 204 p.i., and 3 copies/ml at days 231, 259, and 294 p.i. All data are presented as median ± interquartile range.

To address the virologic impact of BCL-2 inhibition at ARTi, we first evaluated its effect on the kinetics of viral decay (Extended Fig. 2a,b). Plasma viral loads were measured longitudinally, as detailed in the Materials and Methods section. We applied a biphasic decay model to the plasma viral load measurements and found no significant differences between control and venetoclax-alone-treated RMs. In contrast, RMs receiving the combination of venetoclax and anti-CD8α antibodies showed a significantly slower viral decay rate, consistent with our previous observation that CD8α depletion leads to a slower decline in viremia.

**Fig. 3:**
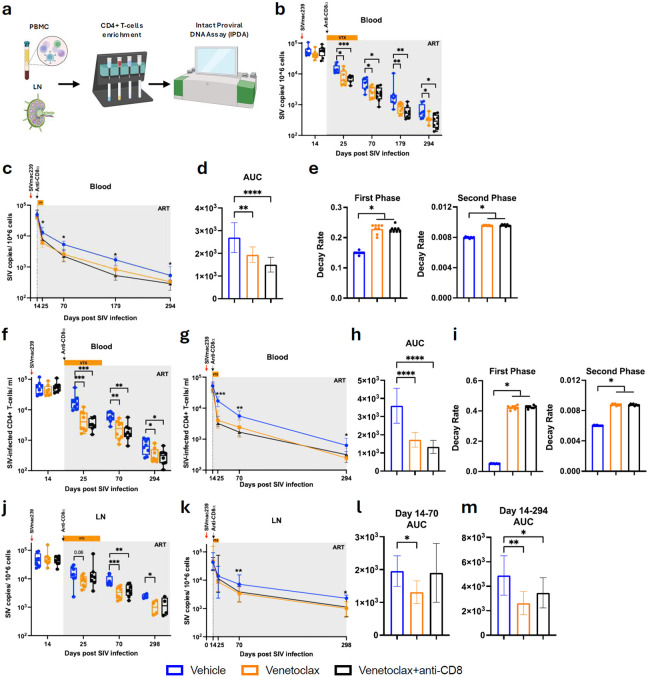
BCL-2 inhibition via venetoclax induces a rapid and sustained reduction of the intact SIV reservoir. a, Experimental workflow. CD4^+^ T cells were isolated from PBMCs and LNs by magnetic enrichment and used to quantify intact SIV proviral DNA with the intact proviral DNA assay (IPDA). b, j, intact SIV DNA copies per 10^6^ CD4^+^ T cells at indicated time points. Shown are individual values with median and 25th–75th percentiles from blood (b) and LNs (j). c, k, Grouped longitudinal kinetics of intact SIV DNA copies per 10^6^ CD4^+^ T cells from blood (c) and LNs (k). Data are shown as median and interquartile range (IQR). d, Area under the curve (AUC) of intact SIV DNA copies per 10^6^ CD4^+^ T cells from day 14 to day 294 p.i. for blood. Shown are mean ± s.d. e, Biphasic decay model of intact SIV DNA copies per 10^6^ CD4^+^ T cells. Shown are individual fits with mean ± s.d. f, Number of CD4^+^ T cells with intact SIV DNA per ml of blood. Individual values are shown with median and 25th–75th percentiles. g, grouped longitudinal kinetics of CD4^+^ T cells with intact SIV DNA per ml of blood, shown as median and IQR. h, AUC of CD4^+^ T cells with intact SIV per ml of blood from day 14 to day 294 p.i., shown as mean ± s.d. i, Biphasic decay model of CD4^+^ T cells with intact SIV DNA per ml of blood. Shown are individual fits with mean ± s.d. l, m, AUC of intact SIV DNA copies per 10^6^ LN CD4^+^ T cells from day 14 to day 70 (l) and day 14 to day 294 (m), shown as mean ± s.d. RMs are color-coded and grouped by treatment arm: vehicle (n = 8), blue; venetoclax (n = 8), orange; venetoclax + anti-CD8 (n = 8), black. Data in panels b, c, f, and g were analyzed using two-sided Mann–Whitney U tests. Data in d and h were analyzed using ordinary one-way ANOVA. Data in e and i were analyzed using the Wald test. P ≤ 0.05, P < 0.01, P < 0.001, P < 0.0001.

Next, to determine the impact of BCL-2 inhibition via venetoclax at ARTi on viral persistence, we quantified the frequency of CD4^+^ T-cells harboring intact SIV DNA in blood and lymphoid tissues using the intact proviral DNA assay (IPDA)^35^ (Fig. 3a and Extended Fig. 3a). The SIV reservoir was measured in sorted CD4^+^ T-cells from PBMCs at baseline (day 14 p.i.), at the end of venetoclax treatment (day 25 p.i.), and during follow-up (days 70, 179, and 294 p.i.). As expected, cross-sectional analysis revealed no differences among experimental arms at the pre-ART baseline. However, we observed a rapid and significant reduction in the levels of cell-associated intact SIV DNA per million CD4^+^ T-cells in venetoclax-treated RMs at the end of the treatment when compared to controls (day 25 p.i.), with this reduction being sustained through days 70, 179, and 294 p.i. (Fig. 3 b,c). To better evaluate the overall impact on viral persistence, we calculated the area under the curve (AUC) of the intact SIV DNA from day 14 to day 294 and found that venetoclax-treated RMs had significantly lower AUC compared to controls (Fig. 3d). To further evaluate reservoir dynamics, we fit a biphasic decay model for intact cell-associated SIV DNA in CD4^+^ T-cells. As compared to control animals, venetoclax increased both the initial decay phase (1.5 fold increase), during which the majority of reservoir clearance occurs, and the slower decay phase (1.2-fold increase) (Fig. 3e).

Venetoclax impacts the size of the reservoir with two main mechanisms, preferentially reducing the frequency of CD4^+^ T-cells harboring intact SIV and reducing the number of blood CD4^+^ T-cells. To integrate both mechanisms, we next evaluated the effect of venetoclax on the absolute number of circulating SIV-infected cells per milliliter of blood. The net reduction in circulating SIV-infected cells observed in venetoclax-treated RMs was even more pronounced, with a substantial drop in intact SIV DNA per milliliter of blood evident immediately after treatment (day 25 p.i.) and which remained significantly reduced through days 70, 179, and 294 p.i. (Fig. 3f, g). Venetoclax-treated RMs also displayed markedly lower AUCs compared to controls (Fig. 3h). Consistent with the cell-normalized reservoir data, biphasic modeling of circulating infected cells confirmed that venetoclax significantly accelerated the fast (8.2-fold increase) and slow (1.5-fold increase)-decaying component (Fig. 3i).

Collectively, these findings demonstrate that venetoclax induces a robust and sustained depletion of SIV-infected cells by selectively enhancing the decay rate of the viral reservoir. This effect is evident both in the frequency of infected cells and, even more strikingly, in the absolute net reduction of circulating infected cells.

In LNs, the SIV reservoir was evaluated in sorted CD4^+^ T-cells at baseline (day 14 p.i.), at the end of venetoclax treatment (day 25 p.i.), and during follow-up at days 70 and 294 p.i. (Fig. 3a; at day 294, sample availability was limited to four RMs per arm). Cross-sectional analysis showed lower levels of intact cell-associated SIV DNA per million CD4^+^ T-cells in venetoclax-treated RMs at day 25 as compared to controls, with differences that become statistically significant at days 70 and 294 p.i. (Fig. 3j,k). We also calculated the area under the curve (AUC) for intact SIV DNA to better evaluate the overall impact on the lymphoid reservoir. When considering RMs up to day 70 (n = 8 per group), the venetoclax-alone treatment group showed significantly lower AUCs than controls (Fig. 3l). When the analysis was extended to day 294, consistently lower AUCs were observed in all venetoclax-treated RMs (Fig. 3m).

In conclusion, venetoclax administration at ARTi effectively and durably disrupts the establishment and maintenance of the SIV reservoir in both blood and lymphoid tissues.

### CD4+BCL-2^+^ cells are reduced by venetoclax, with the remaining ones expressing high level of BCL-2 on a per-cell basis.

**Fig. 4.**
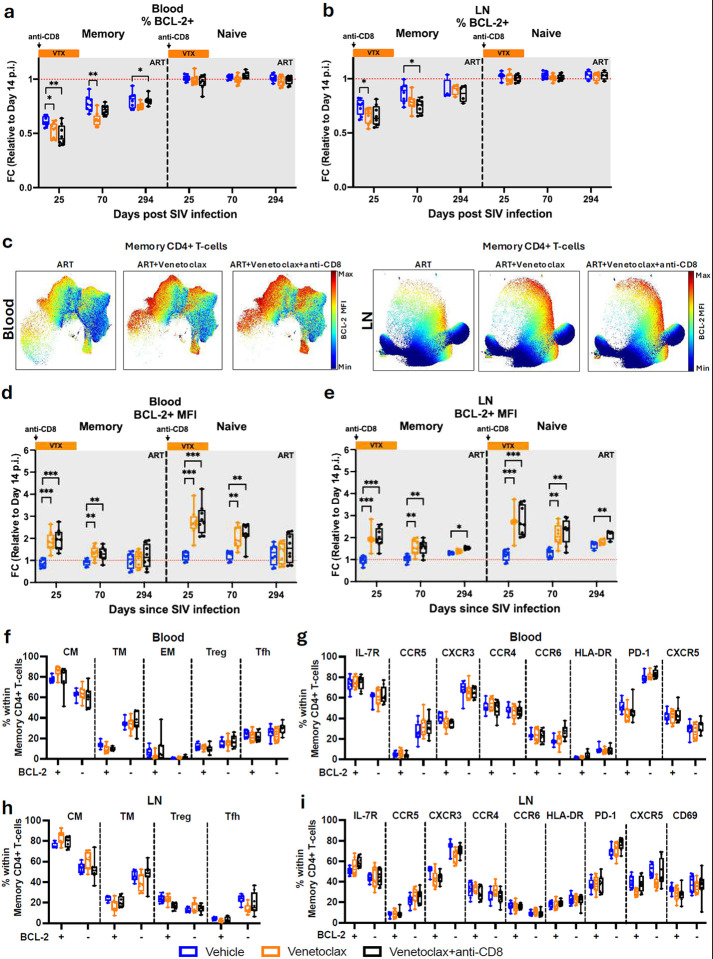
Venetoclax treatment reduces the frequency of CD4^+^BCL-2^+^ T cells, with those persisting showing higher BCL-2 expression on a per-cell basis. a, b, Fold change of the frequency of BCL-2^+^ cells within memory and naïve CD4^+^ T cells relative to day 14 p.i. in blood (a) and LNs (b). c, UMAP visualization of flow cytometry data showing BCL-2 expression patterns in memory CD4^+^ T cells from blood and LNs of vehicle, venetoclax, or venetoclax + anti-CD8 antibody-treated RMs. d, e, Fold change of BCL-2 MFI in BCL2^+^ memory and naïve CD4^+^ T cells relative to day 14 p.i. from blood (d) and LNs (e). f-i, Phenotypic analysis comparing BCL-2^+^ and BCL-2^−^ memory CD4^+^ T cells. Shown is the frequency of T_CM_(CCR7^+^), T_TM_ (CCR7^−^), T_EM_ (CD28−), T_regs_ (CD127-CD25+), and T_fh_ (CXCR5+PD-1+) within BCL-2^+^ and BCL-2^−^ populations in blood (f) and LNs(h). g, i, Frequency of cytokine/chemokine receptor (IL-7R, CCR5, CXCR3, CCR4, CCR6, CXCR5) and activation markers (HLA-DR, PD-1, CD69) expression within BCL-2^+^ and BCL-2^−^ memory CD4^+^ T cells in blood (g) and LNs (i). RMs are color-coded by treatment arm: vehicle (n = 8), blue; venetoclax (n = 8), orange; venetoclax + anti-CD8 (n = 8), black. Data represent median ± 25th and 75th percentiles.

A short treatment with venetoclax was effective in reducing the size of the intact SIV reservoir but was insufficient to fully eliminate it. To better understand the mechanisms underlying cell survival and the persistence of SIV-infected cells following BCL-2 inhibition, we next characterized the immunological features of CD4^+^ T-cells that remained in the blood and LNs after venetoclax treatment. First, we investigated the frequency and expression of BCL-2 in CD4^+^ T-cell subsets at the different experimental points. Following SIV infection (day 14 p.i.), we observed an increased frequency of BCL-2^+^ memory CD4^+^ T-cells with a concomitant loss of BCL-2^−^ memory CD4^+^ T-cells in blood, suggesting a greater survival advantage for BCL-2^+^ cells during SIV infection (Supplementary Fig. 3a). After ARTi, there was a reconstitution of BCL-2^−^ memory CD4^+^ T-cells with a relative decrease in BCL-2^+^ memory CD4^+^ T-cells (Fig. 4a; Supplementary Fig. 3a).

Starting at the end of venetoclax treatment, and sustained over time, treated RMs exhibited lower frequencies of BCL-2^+^ memory (but not naïve) CD4^+^ T-cells compared to controls (Fig. 4a). Similar patterns were observed in the LNs, where venetoclax reduced the frequencies of BCL-2^+^ memory, but not naïve, CD4^+^ T-cells (Fig. 4b; Supplementary Fig. 3b).

Despite the overall reduction in the frequency of BCL-2^+^ memory CD4^+^ T-cells at the end of venetoclax treatment (day 25 p.i.), the remaining BCL-2^+^ memory and naïve CD4^+^ T-cells showed higher per-cell BCL-2 expression levels (MFI) in both blood (Fig. 4c,d; Supplementary Fig. 3c) and LNs (Fig. 4c,e; Supplementary Fig. 3d) when compared to pre-ART baseline (day 14 p.i.) and control RMs. This increase was evident across memory subsets, including central, transitional, and effector cells (Supplementary Fig. 4a–f). Elevated per-cell BCL-2 expression persisted through day 70 p.i. across all subsets in blood and LNs of venetoclax-treated RMs and normalized by day 294 p.i., except for BCL-2^+^ memory and naïve CD4^+^ T-cells from LNs of venetoclax+anti-CD8α -treated RMs, where BCL-2 expression remained slightly elevated.

We then characterized BCL-2^+^ memory CD4^+^ T-cells and compared them to their BCL-2^−^ counterparts at the end of venetoclax treatment, both in blood and LNs (Fig. 4f-i). BCL-2^+^ cells had a higher frequency of central memory (CCR7^+^) and a lower frequency of transitional memory (CCR7^−^) in both compartments. The frequencies of Tregs and Tfh cells did not differ between BCL-2^+^ and BCL-2^−^ populations in blood. However, in LNs, the BCL-2^+^ population showed a lower frequency of Tfh cells and Tregs. We also analyzed the expression of cytokine and chemokine receptors, along with activation markers (Fig. 4g,i). In blood and LNs, BCL-2^+^ cells showed increased IL-7R and decreased PD-1 expression, consistent with enhanced survival potential, and lower levels of CCR5 and CXCR3, suggesting a less activated phenotype. Additionally, BCL-2^+^ cells expressed lower HLA-DR in blood and lower CD69 in LNs compared to BCL-2^−^ cells, further supporting their less activated status. CCR6 was more frequent in BCL-2^+^ cells from LNs, while CCR4 expression did not differ in either tissue. CXCR5 was more abundant in BCL-2^+^ cells from blood but reduced in those from LNs, indicating tissue-specific differences.

In conclusion, our findings prove the efficacy of venetoclax in depleting the key long-lived reservoir of BCL-2^+^ memory CD4^+^ T-cells and show that the remaining BCL-2^+^ memory CD4^+^ T-cells exhibit phenotypic features associated with enhanced survival and reduced activation. These characteristics may promote the resistance of some SIV-infected cells to BCL-2 inhibition and can be targeted in a combinational approach to further decrease the size of the reservoir.

### Survival mechanisms of CD4^+^ T-cells in response to BCL-2 inhibition via venetoclax

**Fig. 5.**
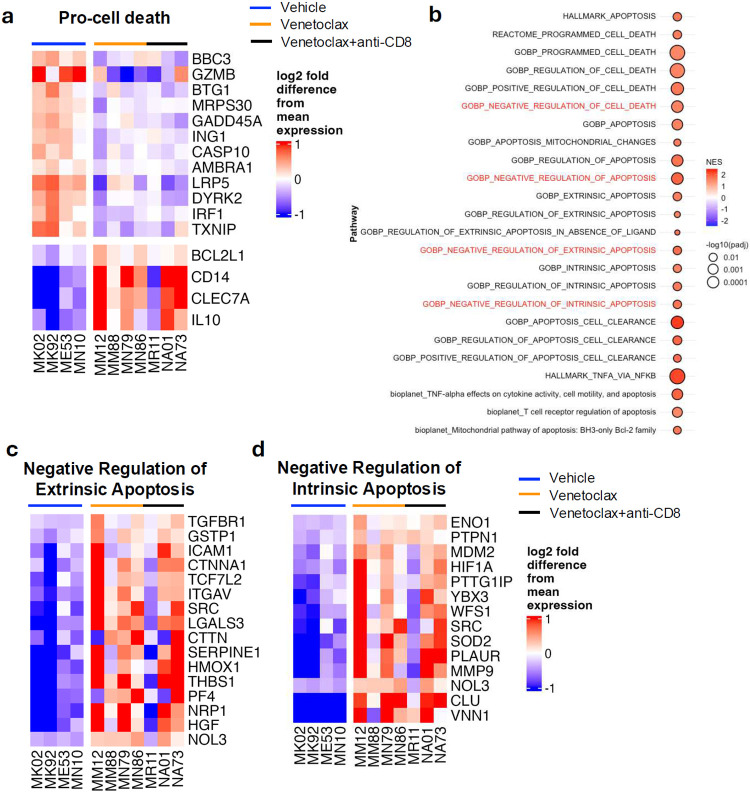
Survival mechanisms of CD4+ T cells are triggered in response to BCL-2 inhibition via venetoclax. a, Heatmap showing log_2_ fold difference from mean expression in CD4^+^ T cells isolated from venetoclax-treated as compared to vehicle-treated RMs at day 25 p.i. Top panel, downregulated pro-cell death genes, bottom panel, upregulated genes associated with venetoclax resistance. b, GSEA results for apoptosis-related gene sets; color indicates normalized enrichment score (NES), circle size indicates p value. c, d, Heatmaps showing genes from the "negative regulation of extrinsic apoptotic signaling" (c) and "negative regulation of intrinsic apoptotic signaling" (d) gene sets; color indicates log_2_ fold difference from mean expression. Analyses were performed on samples from vehicle (n = 4), venetoclax (n = 4), and venetoclax + anti-CD8 (n = 3) treated RMs. Genes with adjusted P <0.05 were included for heatmaps and GSEA results.

To evaluate the mechanisms underlying the persistence of CD4^+^ T-cells following venetoclax treatment, we expanded the transcriptional profiling analysis on purified CD4^+^ T-cells sorted at the end of treatment (day 25 p.i.) from both vehicle- and venetoclax-treated RMs to identify gene expression signatures associated with venetoclax resistance and cell death evasion (Fig. 5a). Venetoclax-treated RMs, as compared to vehicle-treated controls, showed reduced expression of the pro-apoptotic molecule BBC3 (PUMA), whose downregulation has been previously linked to venetoclax resistance in hematologic malignancies^36^. The loss of BBC3 has also been previously associated with a metabolic shift toward increased oxidative phosphorylation, a phenotype linked to resistance to venetoclax. In line with this, venetoclax-treated RMs exhibited enrichment of the oxidative phosphorylation pathway and of mTORC pathway components, known to enhance oxidative phosphorylation (Supplementary Fig. 5a-c). Similarly, GZMB, another pro-apoptotic effector, was downregulated^37^. Additionally, BTG1 and MRPS30, whose inactivation has been linked to venetoclax resistance in leukemia cell lines, exhibited decreased expression^38^. The levels of other positive regulators of apoptosis, including ING1, which promotes BAX expression^39^, and TXNIP, which induces apoptosis under oxidative stress^40^ were also reduced. Finally, we observed upregulation of genes whose heightened expression has been associated with venetoclax resistance such as the anti-apoptotic protein BCL2L1(BCL-xL), the cytokine IL-10, which promotes BCL-2 expression, and the innate immune markers CD14 and CLEC7A. These findings suggest that a subset of CD4^+^ T-cells that persist after venetoclax treatment exhibits transcriptional features consistent with established signatures of venetoclax resistance in cancer diseases.

Further analyses of enriched gene sets in venetoclax-treated RMs revealed, as expected, a significant enrichment of cell death and pro-apoptotic pathways. However, we also observed enrichment of gene sets involved in the negative regulation of both extrinsic and intrinsic apoptotic signaling (Fig. 4b-d, Data set 2). Among the most significantly differentially expressed genes were HMOX1, which protects against TNF-induced apoptosis, and CLU, which inhibits apoptosis by interacting with pro-apoptotic BAX. Notably, NOL3, which downregulates caspase activity, was also found to be upregulated.

In conclusion, CD4^+^ T-cells persisting after venetoclax treatment display transcriptional signatures of venetoclax resistance, characterized by reduced pro-apoptotic gene expression, upregulation of negative regulators of cell death, and metabolic reprogramming toward oxidative phosphorylation.

### CD4^+^ T-Cells from venetoclax-treated RMs remain susceptible to BCL-2 inhibition ex-vivo

We observed that BCL-2 inhibition via venetoclax was effective at inducing cell death; however, regulatory anti-apoptotic and survival mechanisms also emerged. Therefore, we evaluated whether CD4^+^ T-cells retained sensitivity to venetoclax following in vivo treatment, to determine whether prolonged venetoclax exposure could further deplete the SIV reservoir.

**Fig. 6.**
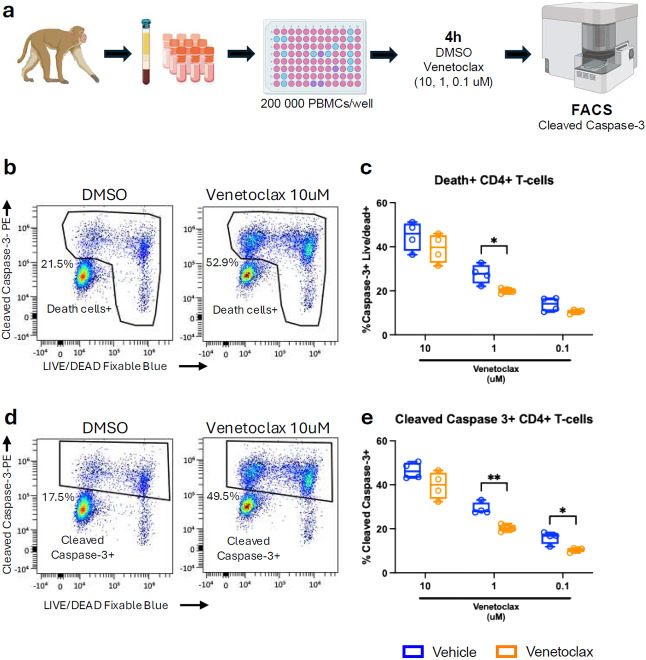
CD4^+^ T cells from venetoclax-treated RMs retain sensitivity to ex vivo BCL-2 inhibition. a, Experimental workflow. Briefly, after thawing, PBMCs from vehicle- and venetoclax-treated RMs were seeded in culture at 200,000 PBMCs per well and treated for 4 hours with DMSO or venetoclax at 10, 1, and 0.1 μM. Samples were then stained and immunophenotyped via flow cytometry. b, Representative gating strategy for CD4^+^ T cells positive for cleaved caspase-3 and Live/Dead from one venetoclax-treated RM, showing DMSO and venetoclax at the 10 μM condition. c, Frequencies of cleaved caspase-3^+^ and Live/Dead^+^ (death+) CD4^+^ T cells, normalized to DMSO, following ex vivo treatment with venetoclax at 10, 1, and 0.1 μM. d, Gating strategy for cleaved caspase-3^+^ CD4^+^ T cells from the same RM and conditions shown in (b). e, Frequency of cleaved caspase-3^+^ CD4^+^ T cells, normalized to DMSO, after ex vivo venetoclax treatment at 10, 1, and 0.1 μM. Analyses were performed on samples from vehicle (n = 4) and venetoclax (n = 4) treated RMs at day 49 p.i. Data are presented as median ± 25th and 75th percentiles. Statistical comparisons were performed using a two-sided Mann–Whitney U test. *P ≤ 0.05, **P < 0.01, ***P < 0.001, ****P < 0.0001.

For this purpose, we isolated PBMCs from 4 vehicle- and 4 venetoclax-alone-treated RMs at day 49 p.i. and cultured them for 4 hours in the presence of DMSO or venetoclax at concentrations of 10, 1, and 0.1 μM. To evaluate the capacity of cells to undergo apoptosis, we stained them with lineage markers to identify CD4^+^ T-cells and evaluated the frequency of cleaved caspase-3^+^ cells and Live/Dead^+^ cells (Fig. 6a). In DMSO-treated samples, we observed a baseline level of spontaneous apoptosis and cell death (Fig. 6b,d) which was subtracted from venetoclax conditions. We first evaluated the total frequencies of death cells, i..e cells that were positive for cleaved caspase-3 and/or live/dead staining (Fig. 6b,c) and observed that these populations were overall comparable between vehicle- and venetoclax-treated RMs. A lower frequency of apoptotic cells was detected only at 1 μM venetoclax treatment, with no significant differences observed when cells were treated with 10 μM and 0.1 μM (Fig 6c). We next analyzed the total frequency of cleaved caspase-3^+^ CD4^+^ T-cells to specifically determine apoptosis sensitivity (Fig 6d,e). At 10 μM venetoclax, no significant differences were observed between vehicle-treated and venetoclax-treated however, venetoclax-treated RMs exhibited a significantly lower frequency of cleaved caspase-3^+^ CD4^+^ T-cells when treated with venetoclax at 1 μM and 0.1 μM (Fig 6e). Altogether, these results indicate that CD4^+^ T-cells persisting after in vivo venetoclax treatment remain susceptible to BCL-2 inhibition, although with a moderate reduction in sensitivity compared to cells from vehicle-treated RMs. These findings support the rationale for extended venetoclax dosing and suggest that combining BCL-2 inhibition with other anti-apoptotic molecules may enhance the clearance of the SIV reservoir.

## Discussion

In this study, we demonstrated, directly in vivo, that BCL-2 inhibition via venetoclax led to a rapid and sustained reduction of the intact SIV reservoir in both blood and LNs of SIV-infected RMs. Mechanistically, venetoclax effectively depleted CD4^+^ T-cell subsets critically contributing to viral persistence by inducing pro-death transcriptional programs. These subsets include central and transitional memory CD4^+^ T-cells and CD4+ T-cells expressing the survival factor BCL-2. Notably, CD4^+^ T-cells that persisted following venetoclax treatment exhibited increased BCL-2 expression on a per-cell basis and upregulation of alternative survival pathways. Although these cells remained responsive to ex vivo venetoclax re-treatment, their apoptotic sensitivity was moderately reduced compared to CD4^+^ T-cells from vehicle-treated animals.

BCL-2 plays a critical role in the survival of infected cells. Following SIV infection, we observed a selective enrichment of BCL-2^+^ CD4^+^ T-cells, likely reflecting resistance to viral cytopathic effects or immune-mediated clearance. These findings are consistent with prior studies showing that memory CD4^+^ T-cells harboring HIV-DNA during ART display transcriptional signatures of resistance to apoptosis, including repression of death receptor and necroptosis pathways^5^. Furthermore, CD4^+^ T-cells harboring HIV-DNA during ART express surface markers associated with enhanced survival signaling, including IL-7R, IL-21R, CD28, OX40, and CD44^3^. Supporting the role of BCL-2 in promoting viral persistence, its ex vivo inhibition via venetoclax reduced the intact HIV reservoir in CD4^+^ T-cells from ART-suppressed PLWH^9,41^; in addition, BCL-2 inhibition in vivo in HIV-infected hu-mice reduced plasma viremia during active infection^4^ and slightly delayed viral rebound after ART interruption^9^.

Our findings in the NHP model of HIV infection support the notion that cells harboring intact proviruses are particularly dependent on BCL-2 for survival, as evidenced by the fact that venetoclax treatment resulted not only in an overall reduction in CD4^+^ T-cell counts but in a specific decline in the frequency of BCL-2^+^ cells and CD4^+^ T-cells containing intact proviruses. Specifically, we observed a rapid - already present at the end of a 10-day treatment period - statistically significant and sustained – up to the latest tested experimental point, at 10 months after venetoclax treatment - reduction in the frequency of blood and LNs CD4^+^ T-cells harboring intact SIV DNA. Since venetoclax reduces both the levels of CD4+ T-cells and their frequency of infection, the reduction in the size of the SIV reservoir was even more evident when considering the absolute quantification of the number of CD4+ T-cells harboring intact SIV DNA. Thus, a short administration of venetoclax in the highly clinically relevant model of SIV infection and ART treatment in RMs was effective in reducing the number of infected cells that can contribute to viral rebound in the absence of ART. Venetoclax is FDA-approved for the treatment of several hematological malignancies^8^, which facilitates its potential repurposing for HIV cure strategies.

Our transcriptional analysis revealed that CD4^+^ T-cells persisting after venetoclax treatment exhibited reduced expression of several key pro-apoptotic genes, including PUMA and GZMB. Consistent with our in vivo finding, ex vivo treatment with venetoclax in cells from PLWH resulted in the selective survival of cells with low PUMA expression^9^. Furthermore, PUMA downregulation has been linked to venetoclax resistance in lymphoma cell lines and chronic lymphocytic leukemia primary cells, where its silencing is mediated by promoter methylation^36^. In those cancer settings, inhibition of DNA methyltransferases restored PUMA expression and re-sensitized resistant cells to venetoclax-induced apoptosis. These findings suggest that epigenetic suppression of PUMA may contribute to the persistence of infected CD4^+^ T-cells following BCL-2 inhibition. Therapeutic strategies that restore or enhance PUMA expression may therefore improve the effectiveness of venetoclax in targeting and eliminating the viral reservoir.

The frequency of CD4^+^ T-cells expressing BCL-2 was reduced in RMs treated with venetoclax, supporting the ability of this therapeutic strategy to reduce even the most difficult to eliminate viral reservoir. However, CD4^+^ T-cells from venetoclax-treated animals exhibited higher levels of BCL-2 expression on a per-cell basis at the end of the treatment when compared to vehicle-treated controls. This mirrors findings in leukemic B-cells from patients with chronic lymphocytic leukemia (CLL), where the surviving B cells show higher BCL-2 expression following venetoclax treatment^42,43^. In CLL, this increased BCL-2 expression contributes to resistance upon ex vivo re-treatment with venetoclax. In our study, CD4^+^ T-cells from venetoclax-treated SIV-infected RMs remained susceptible to ex vivo re-treatment, although higher doses of venetoclax were required compared to vehicle controls. This reduced sensitivity may result, at least partially, by BCL-2 upregulation following in vivo treatment. We also observed increased expression of the anti-apoptotic molecule BCL-xL (BCL2L1) in venetoclax-treated animals, consistent with findings from CLL patients after venetoclax therapy. In the clinical setting of CLL, venetoclax is typically administered over several months and clinical trials are currently evaluating its combination with agents targeting other anti-apoptotic proteins, such as BCL-xL. Considering we see that CD4^+^ T-cells surviving our short in vivo venetoclax treatment are (i) still susceptible to cell death when treated with venetoclax ex-vivo and (ii) characterized by upregulation of BCL-xL and other pro-survival molecules, we propose that prolonged venetoclax administration and its combination with drugs targeting those additional anti-apoptotic pathways will further enhance the clearance of the SIV reservoir. Our transcriptional data have identified several targetable pathways that is worth exploring for synergy with venetoclax in future studies aimed at HIV cure.

Of note, the main virologic and immunologic readouts of our study were similar between SIV-infected RMs treated with venetoclax or venetoclad and CD8 depletion. This data suggests that the pro-apoptotic activity of venetoclax does not require the prolonged on-ART viremia induced by CD8 depletion (this study and ^10^) or the presence of cytolytic CD8^+^ T-cells, at least when BCL-2 is inhibited at ART initiation. Based on this study design, we cannot exclude the need for cytolytic CD8+ T-cells or viral reactivation for venetoclax to be effective in reducing the SIV or HIV viral reservoir if used in fully suppressed NHP or humans. New studies have been designed to address this specific question in SIV-infected RMs on long-term ART treated with venetoclax and LRA strategies, and a clinical trial is currently evaluating the safety and impact of venetoclax on the HIV reservoir in ART-suppressed PLWH.

In conclusion, this study presents the first evaluation of venetoclax administration at ARTi in an NHP model of SIV infection. These findings describe the efficacy of a short BCL-2 inhibition in durably limiting SIV persistence, provide mechanistic insights for the venetoclax-induced death of CD4+ T-cells that critically contribute to viral persistence, and support the rationale for prolonged venetoclax dosing and for combining BCL-2 inhibition with agents targeting additional anti-apoptotic molecules to further enhance clearance of the viral reservoir in HIV cure strategies.

## Methods

### Study approval

All animal experiments were conducted in accordance with the Animal Welfare Act and the NIH Guide for the Care and Use of Laboratory Animals. The animal facilities at the Emory National Primate Research Center are accredited by the U.S. Department of Agriculture and the Association for Assessment and Accreditation of Laboratory Animal Care International. This study was reviewed and approved by Emory University’s Institutional Animal Care and Use Committee (IACUC; Protocol #PROT202100086). All procedures were performed under anesthesia, with appropriate pain management as necessary. Proper measures were taken to minimize animal suffering.

### Animals, SIV infection and ART

A total of 24 Indian-origin Macaca mulatta were included in this study. Animal identifiers, age at the time of infection, treatment arm assignment, and Mamu*A01 genotype are provided in Supplementary Table 1. All RMs were Mamu*B08- and Mamu*B17-. RMs were infected IV with 10,000 IU of barcoded SIV_mac239M_. SIV_mac239M_ was provided by Brandon Keele. Triple-combination ART was started on day 14 p.i. and consisted of TDF (5.1 mg kg^−1^ d^−1^, Gilead), DTG (2.5 mg kg^−1^ d^−1^, ViiV), and emtricitabine FTC (40 mg kg^−1^ d^−1,^ Gilead). RMs were stratified into three experimental arms: vehicle, venetoclax, or venetoclax combined with anti-CD8α depletion.

### Venetoclax formulation and administration

For SC administration, venetoclax powder (catalog no. S8048, Selleckchem) was dissolved in a vehicle solution composed of 25% dimethyl sulfoxide (DMSO; catalog no. NC9629398, Protide Pharmaceuticals), 10% Tween 80 (catalog no. S6702, Selleckchem), and 65% polyethylene glycol 300 (PEG300; catalog no. 6704, Selleckchem). The formulation was freshly prepared each day immediately before administration. For OG administration, venetoclax was used in the form of Venclexta (AbbVie). Four 100 mg tablets were crushed using a mortar and pestle and subsequently dissolved in Ensure^®^ before dosing. A total of 16 RMs were treated with venetoclax using the following regimens. Four RMs received ten SC doses at 20 mg/kg; . Another four received five SC doses at 20 mg/kg followed by five SC doses at 10 mg/kg, except one animal that received only three of the 10 mg/kg doses. The remaining eight RMs underwent two consecutive dosing cycles, each consisting of two SC doses at 20 mg/kg followed by three OG doses of 400 mg Venclexta (the FDA-approved formulation of venetoclax), co-administered with the CYP3A inhibitor ketoconazole (10 mg/kg). A two-day interval was maintained between the fifth and sixth doses of venetoclax for all RMs.

### Anti-CD8α depletion antibody

The anti-CD8α-depleting antibody MT807R1 was obtained from the Non-Human Primate Reagent Resource and administered as a single subcutaneous dose of 50 mg/kg at the time of ARTi.

### Measurement of Venetoclax in plasma

Venetoclax was extracted from non-human primate samples by protein precipitation with methanol containing the stable, isotopically-labeled internal standard, ^2^H_7_-venetoclax. These samples were then vortexed, centrifuged, and transferred to a 96-well plate for analysis. Plasma extracts were analyzed by LC-MS/MS under gradient conditions on a Waters Atlantis T3 (50x2.1mm, 3μm particle size) analytical column with 0.1% formic acid in water (mobile phase A) and 0.1% formic acid in acetonitrile (mobile phase B) with detection on an AB Sciex API-5000 triple quadrupole mass spectrometer. The calibration range for the assay was 2.00-10,000ng/mL. Calibration standards and quality control samples were within 20% of nominal concentrations.

### Samples collection and processing

Blood was collected on days 0, 14, 15, 17, 21, 25, 28, 49, 56, 70, 84, 112, 142, 179, 204, 231, 260, and 294 post-infection (p.i.), while lymph node (LNs) biopsies were obtained on days 14, 25, 70, and 298 p.i. EDTA-anticoagulated blood was used for complete blood counts, and plasma was separated by centrifugation within 1 hour of collection. PBMCs were isolated via density gradient centrifugation (Ficoll-Paque Premium, GE Healthcare). For LNs sampling, the overlying skin at the axillary or inguinal site was surgically prepared. An incision was made in the skin, and the LNs was exposed by blunt dissection and excised over clamps. Harvested LNs were sectioned with a sterile scalpel, dissociated in RPMI 1640 medium supplemented with 10% heat-inactivated fetal bovine serum (FBS, Gemini Bio), 100 U/ml penicillin, and 100 μg/ml streptomycin, and then filtered through a 100-μm strainer to isolate mononuclear cells. Single-cell suspensions were cryopreserved in 10% DMSO in FBS.

### Flow cytometry

PBMC and mononuclear cells from LNs were stained using the following antibodies at predetermined optimal concentrations: anti-CCR7-BB700 (clone 3D12, catalog no. 566437), anti-CXCR3-BV421 (clone 1C6/CXCR3, catalog no. 562558), anti-CCR4-PE-CF594 (clone 1G1, catalog no. 565391), anti-CD3-BUV395 (clone SP34-2, catalog no. 564117), anti-CD8-BUV496 (clone RPA-T8, catalog no. 612942), anti-NHP CD45-BUV563 (clone D058-1283, catalog no. 741414), anti-CD28-BUV737 (clone CD28.2, catalog no. 612815), anti-CD69-BUBCL-2V805 (clone FN50, catalog no. 748763), anti-BCL-2-AF647 (clone Bcl-2/100, catalog no. 563600), anti-active caspase-3 (clone C92-605.rMAb, catalog no. 570185)(all from BD Bioscience); anti-CD95-BV605 (clone DX2, catalog no. 305628), anti-HLA-DR-BV650 (clone L243, catalog no. 307650), anti-CD25-BV711 (clone BC96, catalog no. 302636), anti-PD-1-BV785 (clone EH12.2H7, catalog no. 329930), anti-CCR6-PE (clone G034E3, catalog no. 353410), anti-CD4-APC-Cy7 (clone OKT4, catalog no. 317418)(all from Biolegend); anti-CD127-PE-Cy5 (clone eBioRDR5, catalog no. 15-1278-42), anti-CD185-PE-Cy7 (clone MU5UBEE, catalog no. 25-9185-42) )(all from Thermo Fisher Scientific). To discriminate viable from non-viable cells, samples were stained with the dye BD Horizon^™^ Fixable Viability Stain 700 (catalog no. 564997, BD Bioscience) or LIVE/DEAD^™^ Fixable Blue Dead Cell Stain Kit, for UV excitation(catalog no. L34962). Intracellular staining to evaluate BCL-2 expression and cleaved caspase-3 was performed using the BD Cytofix/Cytoperm^™^ Fixation/Permeabilization Kit (catalog no. 554714, BD Bioscience). Flow cytometry acquisition was performed on a FACSymphony A5 (BD Biosciences) driven by FACS DiVa software for tissue samples or a Cytek Aurora (Cytek) run by SpectroFlo software for ex vivo experiments. Flow cytometry data was analyzed using the software FlowJo version 10 (BD) and OMIQ(Dotmatics). Gating strategies are shown in Supplementary Figures 6 and 7.

### CD4+ T-cell Isolation

Total CD4+ T-cells were isolated from PBMCs by positive selection using the NHP CD4+ T Cell Isolation Kit (catalog no. 130-092-144, Miltenyi Biotech), on an LS column (catalog no. 130-042-401, Miltenyi Biotech) following manufacturer’s specifications. The sorted cells were subsequently used for either IPDA or bulk RNA-seq analysis.

### Viral load RNA

A hybrid qRT-PCR assay was conducted to quantify SIV RNA copies per milliliter of EDTA plasma (i.e., viral load), as previously described^44^. The assay has a detection limit of 15 or 3 copies/mL. Primer and probe sequences are provided in Supplementary Table 2.

### Quantification of IPDA

IPDA was conducted on sorted CD4^+^ T-cells by Accelevir Diagnostics using a method analogous to the HIV-1 IPDA^35^. Droplets were analyzed using a QX100 Droplet Reader (Bio-Rad). Briefiy, extracted DNA was combined with ddPCR Supermix for Probes (Bio-Rad), along with primers and probes for the detection of 2LTR circles and internal pol or env regions. Cell counts were determined by targeting the rhesus macaque RPP30 gene, which enabled the filtering out of samples with excessive DNA shearing. ddPCR readouts were analyzed using QuantaSoft Analysis-Pro software (Bio-Rad). Intact and defective genome copy numbers were normalized to copies per million cells, as determined by the number of RPP30 copies.

### Viral decay rate calculation

To assess viral decay in blood plasma, pVL data for each animal was fit to a biphasic and triphasic decay model, with AIC scores used for model selection, resulting in 8 animals with biphasic decays and 16 animals with triphasic decays. The triphasic decay formula is $VL=a_{1}e^{r_{1}t}+a_{2}e^{r_{2}t}+a_{3}e^{r_{3}t}$.

We fit a biphasic decay model for(log-transformed) intact cell-associated SIV DNA in CD4^+^ T-cells and the absolute number of circulating SIV-infected cells per milliliter of blood using the formula:

(Eq. 1) $$y=a_{1}e^{r_{1}t}+a_{2}e^{r_{2}t},$$

where $r_{1}$, $r_{2}$ are the rates of first and second phase of decay, respectively and $a_{1}$, $a_{2}$ are population size estimates.

Fits are performed using non-linear mixed effects model (NLME) on Monolix 2024R1 (Lixoft) with random effects on individual animals, and the treatment group as a covariate on $r_{1}$ and $r_{2}$. The individual model for estimating the parameters then is:

(Eq. 2) $$\log(a_{1})=\log\;(a_{1_{pop}})+\eta_{a_{1}}\;\log(a_{2})=\log\;(a_{2_{pop}})+\eta_{a_{2}}\;\log(r_{1})=\log\left(r_{1_{pop}}\right)+\beta_{r_{1}Treatment\;group}[Treatment\;group=Treatment]+\eta_{r_{1}}\;\log(r_{2})=\log\left(r_{2_{pop}}\right)+\beta_{r_{2}Treatment\;group}[Treatment\;group=Treatment]+\eta_{r_{2}}$$

The Wald Test was used to assess whether treatment had a significant effect on the decay rates (e.g. whether $\beta_{r_{1}}$ and $\beta_{r_{2}}$ are significantly different from zero).

### Bulk RNA-seq library and sequencing

CD4+ T-cells were lyse in 350 μL of Buffer RLT and then extracted using the RNeasy Mini / kit (Qiagen) with on-column DNase digestion. RNA quality was assessed using a TapeStation 4200 (Agilent) and then ten nanograms of total RNA was used as input for cDNA synthesis using the SMART-Seq v4 Ultra Low Input RNA kit (Takara Bio) according to the manufacturer’s instructions. Ampliefid cDNA was fragmented and appended with dual-indexed barcodes using the Nextera XT DNA Library Preparation kit (Illumina). Libraries were validated by capillary electrophoresis on a TapeStation 4200 (Agilent), pooled at equimolar concentrations, and sequenced with PE100 reads on an Illumina NovaSeq 6000, yielding ~21 million reads per sample on average.

### Bulk RNA-seq analysis

Alignment was performed using STAR version 2.7.9a and transcripts were annotated using a composite genomic assembly and annotation of the Mmul10 Indian rhesus macaque and SIVsm804ECL757 (Accession MF370842). Transcript abundance estimates were calculated internal to the STAR aligner using the algorithm of htseq-count. DESeq2 was used for normalization and differential expression analysis using the Wald test. Functional enrichment was performed using the Gene Set Enrichment Analysis (GSEA) method implemented in the fgsea R package against the Hallmark and Canonical Pathways collections from the Molecular Signatures Database (MSigDB)^45-50^.

### Statistical analysis

Statistical analyses were performed with GraphPad Prism 10 software (GraphPad Software, Inc., La Joya, CA, USA). Statistical test used to compare treatment groups were two-sided Mann–Whitney U tests, and ordinary one-way ANOVA with Fisher’s LSD test. P values ≤ 0.05 were considered statistically significant.

## Extended Data

## Supplementary Material

1

This is a list of supplementary files associated with this preprint. Click to download.

PaiardinilabsupplementaryFiguresRF3venetoclax.pdf

DifferentialexpressedgenesvenetoclaxvsARTtreatedRMsday25and294.xlsx

GSEAvenetoclaxvsARTtreatedRMsday25.xlsx

DataaccessibilityforreviewersA143343.docx

PaiardinilabsupplementaryFiguresRF3venetoclax.pdf

NMEDA143343rs.pdf

## Data Availability

Bulk RNA-seq data have been deposited in the NCBI GEO database under accession number GSE301433. All other data in this article is available upon reasonable request.

## Code Availability

The custom code used in this manuscript is available from the corresponding author upon reasonable request.

## References

[1] Wojciechowski S. Bim/Bcl-2 balance is critical for maintaining naive and memory T cell homeostasis. J Exp Med 204, 1665–1675 (2007). 10.1084/jem.20070618 17591857 PMC2118628

[2] Kuo H. H. Anti-apoptotic Protein BIRC5 Maintains Survival of HIV-1-Infected CD4(+) T Cells. Immunity 48, 1183–1194 e1185 (2018). 10.1016/j.immuni.2018.04.004 29802019 PMC6013384

[3] Sun W. Phenotypic signatures of immune selection in HIV-1 reservoir cells. Nature 614, 309–317 (2023). 10.1038/s41586-022-05538-8 36599977 PMC9908552

[4] Ren Y. BCL-2 antagonism sensitizes cytotoxic T cell-resistant HIV reservoirs to elimination ex vivo. J Clin Invest 130, 2542–2559 (2020). 10.1172/JCI132374 32027622 PMC7191002

[5] Clark I. C. HIV silencing and cell survival signatures in infected T cell reservoirs. Nature 614, 318–325 (2023). 10.1038/s41586-022-05556-6 36599978 PMC9908556

[6] Chandrasekar A. P., Cummins N. W. & Badley A. D. The Role of the BCL-2 Family of Proteins in HIV-1 Pathogenesis and Persistence. Clin Microbiol Rev 33 (2019). 10.1128/CMR.00107-19 PMC6822993 31666279

[7] Chandrasekar A. P. The BCL-2 Inhibitor Venetoclax Augments Immune Effector Function Mediated by Fas Ligand, TRAIL, and Perforin/Granzyme B, Resulting in Reduced Plasma Viremia and Decreased HIV Reservoir Size during Acute HIV Infection in a Humanized Mouse Model. J Virol 96, e0173022 (2022). 10.1128/jvi.01730-22 36448802 PMC9769373

[8] Croce C. M. The BCL-2 protein family: from discovery to drug development. Cell Death Differ (2025). 10.1038/s41418-025-01481-z PMC12325696 40204952

[9] Arandjelovic P. Venetoclax, alone and in combination with the BH3 mimetic S63845, depletes HIV-1 latently infected cells and delays rebound in humanized mice. Cell Rep Med 4, 101178 (2023). 10.1016/j.xcrm.2023.101178 37652018 PMC10518630

[10] Statzu M. CD8(+) lymphocytes do not impact SIV reservoir establishment under ART. Nat Microbiol 8, 299–308 (2023). 10.1038/s41564-022-01311-9 36690860 PMC9894752

[11] Guan J. J. DRAM1 regulates apoptosis through increasing protein levels and lysosomal localization of BAX. Cell Death Dis 6, e1624 (2015). 10.1038/cddis.2014.546 25633293 PMC4669745

[12] Moyer A., Tanaka K. & Cheng E. H. Apoptosis in Cancer Biology and Therapy. Annu Rev Pathol 20, 303–328 (2025). 10.1146/annurev-pathmechdis-051222-115023 39854189

[13] Heim V. J., Stafford C. A. & Nachbur U. NOD Signaling and Cell Death. Front Cell Dev Biol 7, 208 (2019). 10.3389/fcell.2019.00208 31632962 PMC6783575

[14] Vanamee E. S. & Faustman D. L. On the TRAIL of Better Therapies: Understanding TNFRSF Structure-Function. Cells 9 (2020). 10.3390/cells9030764 PMC7140660 32245106

[15] van Loo G. & Bertrand M. J. M. Death by TNF: a road to inflammation. Nat Rev Immunol 23, 289–303 (2023). 10.1038/s41577-022-00792-3 36380021 PMC9665039

[16] Zeng L. Death receptor 6 induces apoptosis not through type I or type II pathways, but via a unique mitochondria-dependent pathway by interacting with Bax protein. J Biol Chem 287, 29125–29133 (2012). 10.1074/jbc.M112.362038 22761420 PMC3436565

[17] van der Weyden C. A., Pileri S. A., Feldman A. L., Whisstock J. & Prince H. M. Understanding CD30 biology and therapeutic targeting: a historical perspective providing insight into future directions. Blood Cancer J 7, e603 (2017). 10.1038/bcj.2017.85 28885612 PMC5709754

[18] Yuan J. & Ofengeim D. A guide to cell death pathways. Nat Rev Mol Cell Biol 25, 379–395 (2024). 10.1038/s41580-023-00689-6 38110635

[19] Bertheloot D., Latz E. & Franklin B. S. Necroptosis, pyroptosis and apoptosis: an intricate game of cell death. Cell Mol Immunol 18, 1106–1121 (2021). 10.1038/s41423-020-00630-3 33785842 PMC8008022

[20] Liu J. cGAS-STING, inflammasomes and pyroptosis: an overview of crosstalk mechanism of activation and regulation. Cell Commun Signal 22, 22 (2024). 10.1186/s12964-023-01466-w 38195584 PMC10775518

[21] Fu J., Schroder K. & Wu H. Mechanistic insights from inflammasome structures. Nat Rev Immunol 24, 518–535 (2024). 10.1038/s41577-024-00995-w 38374299 PMC11216901

[22] Chu B. ALOX12 is required for p53-mediated tumour suppression through a distinct ferroptosis pathway. Nat Cell Biol 21, 579–591 (2019). 10.1038/s41556-019-0305-6 30962574 PMC6624840

[23] Liu J., Kang R. & Tang D. Signaling pathways and defense mechanisms of ferroptosis. FEBS J 289, 7038–7050 (2022). 10.1111/febs.16059 34092035

[24] Kang K., Park C. & Chan F. K. Necroptosis at a glance. J Cell Sci 135 (2022). 10.1242/jcs.260091 PMC10658918 36098620

[25] Nailwal H. & Chan F. K. Necroptosis in anti-viral inflammation. Cell Death Differ 26, 4–13 (2019). 10.1038/s41418-018-0172-x 30050058 PMC6294789

[26] Liu T. Toll-like receptor 4-mediated necroptosis in the development of necrotizing enterocolitis. Pediatr Res 91, 73–82 (2022). 10.1038/s41390-021-01457-y 33731807 PMC8770135

[27] Choi Y. E. The E3 ubiquitin ligase cIAP1 binds and ubiquitinates caspase-3 and -7 via unique mechanisms at distinct steps in their processing. J Biol Chem 284, 12772–12782 (2009). 10.1074/jbc.M807550200 19258326 PMC2676007

[28] Abbas H. Apoptosis Inhibitor 5: A Multifaceted Regulator of Cell Fate. Biomolecules 14 (2024). 10.3390/biom14010136 PMC10813780 38275765

[29] Courtois L. IL-7 receptor expression is frequent in T-cell acute lymphoblastic leukemia and predicts sensitivity to JAK inhibition. Blood 142, 158–171 (2023). 10.1182/blood.2022017948 37023368

[30] Shaw L. A. Id3 expression identifies CD4(+) memory Th1 cells. Proc Natl Acad Sci U S A 119, e2204254119 (2022). 10.1073/pnas.2204254119 35858332 PMC9303986

[31] Kim J. W., Ferris R. L. & Whiteside T. L. Chemokine C receptor 7 expression and protection of circulating CD8+ T lymphocytes from apoptosis. Clin Cancer Res 11, 7901–7910 (2005). 10.1158/1078-0432.CCR-05-1346 16278415

[32] Priem D., van Loo G. & Bertrand M. J. M. A20 and Cell Death-driven Inflammation. Trends Immunol 41, 421–435 (2020). 10.1016/j.it.2020.03.001 32241683

[33] Halder S. K., Beauchamp R. D. & Datta P. K. Smad7 induces tumorigenicity by blocking TGF-beta-induced growth inhibition and apoptosis. Exp Cell Res 307, 231–246 (2005). 10.1016/j.yexcr.2005.03.009 15922743

[34] Luo Y. BCL10 in cell survival after DNA damage. Clin Chim Acta 495, 301–308 (2019). 10.1016/j.cca.2019.04.077 31047877

[35] Bender A. M. The Landscape of Persistent Viral Genomes in ART-Treated SIV, SHIV, and HIV-2 Infections. Cell Host Microbe 26, 73–85 e74 (2019). 10.1016/j.chom.2019.06.005 31295427 PMC6724192

[36] Thomalla D. Deregulation and epigenetic modification of BCL2-family genes cause resistance to venetoclax in hematologic malignancies. Blood 140, 2113–2126 (2022). 10.1182/blood.2021014304 35704690 PMC10653032

[37] Boivin W. A., Cooper D. M., Hiebert P. R. & Granville D. J. Intracellular versus extracellular granzyme B in immunity and disease: challenging the dogma. Lab Invest 89, 1195–1220 (2009). 10.1038/labinvest.2009.91 19770840 PMC7102238

[38] Zhang L. CRISPR screen of venetoclax response-associated genes identifies transcription factor ZNF740 as a key functional regulator. Cell Death Dis 15, 627 (2024). 10.1038/s41419-024-06995-x 39191721 PMC11350041

[39] Bose P. ING1 induces apoptosis through direct effects at the mitochondria. Cell Death Dis 4, e788 (2013). 10.1038/cddis.2013.321 24008732 PMC3789179

[40] Pan M., Zhang F., Qu K., Liu C. & Zhang J. TXNIP: A Double-Edged Sword in Disease and Therapeutic Outlook. Oxid Med Cell Longev 2022, 7805115 (2022). 10.1155/2022/7805115 35450411 PMC9017576

[41] Kadiyala G. N. Differential susceptibility of cells infected with defective and intact HIV proviruses to killing by obatoclax and other small molecules. AIDS 38, 1281–1291 (2024). 10.1097/QAD.0000000000003908 38626436 PMC11216394

[42] Haselager M. V. Changes in Bcl-2 members after ibrutinib or venetoclax uncover functional hierarchy in determining resistance to venetoclax in CLL. Blood 136, 2918–2926 (2020). 10.1182/blood.2019004326 32603412

[43] Jayappa K. D. Extrinsic interactions in the microenvironment in vivo activate an antiapoptotic multidrug-resistant phenotype in CLL. Blood Adv 5, 3497–3510 (2021). 10.1182/bloodadvances.2020003944 34432864 PMC8525241

[44] Li H. Envelope residue 375 substitutions in simian-human immunodeficiency viruses enhance CD4 binding and replication in rhesus macaques. Proc Natl Acad Sci U S A 113, E3413–3422 (2016). 10.1073/pnas.1606636113 27247400 PMC4914158

[45] Dobin A. STAR: ultrafast universal RNA-seq aligner. Bioinformatics 29, 15–21 (2013). 10.1093/bioinformatics/bts635 23104886 PMC3530905

[46] Zimin A. V. A new rhesus macaque assembly and annotation for next-generation sequencing analyses. Biol Direct 9, 20 (2014). 10.1186/1745-6150-9-20 25319552 PMC4214606

[47] Anders S., Pyl P. T. & Huber W. HTSeq--a Python framework to work with high-throughput sequencing data. Bioinformatics 31, 166–169 (2015). 10.1093/bioinformatics/btu638 25260700 PMC4287950

[48] Love M. I., Huber W. & Anders S. Moderated estimation of fold change and dispersion for RNA-seq data with DESeq2. Genome Biol 15, 550 (2014). 10.1186/s13059-014-0550-8 25516281 PMC4302049

[49] Subramanian A. Gene set enrichment analysis: a knowledge-based approach for interpreting genome-wide expression profiles. Proc Natl Acad Sci U S A 102, 15545–15550 (2005). 10.1073/pnas.0506580102 16199517 PMC1239896

[50] Liberzon A. Molecular signatures database (MSigDB) 3.0. Bioinformatics 27, 1739–1740 (2011). 10.1093/bioinformatics/btr260 21546393 PMC3106198

