# Supplementary material for: BCL-2 Inhibition via Venetoclax at ART Initiation Induces Long-Term Reduction of the Intact SIV Reservoir
Source: Res Sq. 2025 Jul 14:rs.3.rs-7060088. Preprint. [Version 1] doi: 10.21203/rs.3.rs-7060088/v1 (PMC12288516; doi:10.21203/rs.3.rs-7060088/v1)
Supplement: 1 [file NIHPPrs7060088v1-supplement-1.pdf]

Supplementary Table 1. Rhesus macaques characteristics

Animal ID	Sex	Age (Months)	Mamu*A01 genotype	Treatment
MK02	Male	53	-	ART
MK92	Male	53	+	ART
MM12	Male	52	-	ART+Venetoclax
MM88	Male	52	+	ART+Venetoclax
MR11	Male	52	-	ART+Venetoclax+anti-CD8
NA01	Male	51	-	ART+Venetoclax+anti-CD8
ME53	Male	64	-	ART
MN10	Male	52	-	ART
MN79	Male	52	-	ART+Venetoclax
MN86	Male	52	-	ART+Venetoclax
NA11	Male	51	-	ART+Venetoclax+anti-CD8
NA73	Male	50	-	ART+Venetoclax+anti-CD8
MN70	Male	68	+	ART+Venetoclax+anti-CD8
MP03	Male	68	-	ART+Venetoclax
MR79	Male	68	-	ART
NA29	Male	67	-	ART+Venetoclax
RGd21	Male	44	-	ART+Venetoclax+anti-CD8
RJb21	Male	44	-	ART
RHj21	Male	43	-	ART+Venetoclax
RMw20	Male	45	-	ART+Venetoclax+anti-CD8
RNe21	Male	44	-	ART+Venetoclax
RPw20	Male	45	-	ART
RUf21	Male	44	-	ART
RUz20	Male	45	-	ART+Venetoclax+anti-CD8

Supplementary Table 2. Primer for qRT-PCR assays.

Assay	Primer name		Sequence
Plasma viremia	Forward Primer	sGAG21	5'-GTCTGCGTCAT(dP)TGGTGCATTC-3'
	Reverse Primer	sGAG22	5' -CACTAG(dK)TGTCTCTGCACTAT(dP)TGTTTTG-3'
	Probe	psGAG23	5' -FAM-CTTC(dP)TCAGT(dK)TGTTTCACTTTCTCTTCTGCG-BHQ 1-3'
